# Banti’s Disease

**Published:** 1918-08

**Authors:** 


**Banti's Disease.** Chas. Norris et al., Am. Jour. Med. Sci., Dec. 1917, consider that this is neither clinically nor anatomically an entity, the argument being obvious to any one who is inclined to doubt the propriety either of starting with a peculiar and rare case to find other cases to match, or with a definition depending on the coincidence of unusual conditions, and of giving his own or a technical name to the symptom complex. It seems, however, that they ascribe rather too much importance to syphilis as an explanation of the symptom complex of Banti's disease.

